# Three-stranded antiviral attack

**DOI:** 10.7554/eLife.02369

**Published:** 2014-03-04

**Authors:** Jenish R Patel, Adolfo García-Sastre

**Affiliations:** 1 **Jenish R Patel** is in the Department of Microbiology, and the Global Health and Emerging Pathogens Institute, Icahn School of Medicine at Mount Sinai, New York, United Statesjenish.patel@mssm.edu; 2 **Adolfo García-Sastre** is in the Department of Microbiology, Department of Medicine, and the Global Health and Emerging Pathogens Institute, Icahn School of Medicine at Mount Sinai, New York, United StatesAdolfo.Garcia-Sastre@mssm.edu

**Keywords:** MAVS, innate immunity, prion-like filaments, cryoEM reconstruction, three-stranded filaments, immune response, Human, viruses

## Abstract

Mitochondrial antiviral signalling proteins form an intricate three-stranded helical filament that has a central role in the response of cells to viruses.

**Related research article** Xu H, He X, Zheng H, Huang LJ, Hou F, Yu Z, de la Cruz MJ, Borkowski B, Zhang X, Chen ZJ, Jiang Q-X. 2014. Structural basis for the prion-like MAVS filaments in antiviral innate immunity. *eLife* **3**:e01489. doi: 10.7554/eLife.01489

**Image** MAVS proteins form a three-stranded filament that is important in viral defence

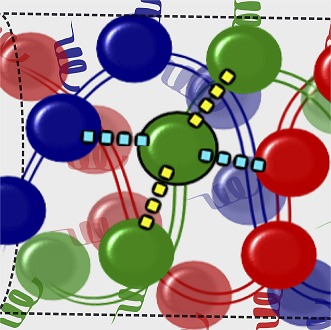

War is constantly being waged between cells and viruses. Viruses need to get inside cells and hijack their systems in order to reproduce, while cells have an immune response to detect and eliminate viruses and other pathogens. The first line of defence against viruses is the innate immune response, which strikes indiscriminately at any pathogen it encounters. A pathway called the type-I interferon (IFN-I) signalling pathway has a central role in beginning the innate immune response. This pathway starts with the viruses being detected by sensors inside the cells, and ends with the production of interferons—proteins that create antiviral conditions around the virus-infected cells.

RNA viruses can be detected by a number of biological sensors, including RIG-I and MDA5, which tend to form large complexes with viral RNA (Yoneyama and Fujita, 2009; Berke and Modis, 2012; Peisley et al., 2011; Patel et al., 2013). These complexes then engage with proteins called mitochondrial antiviral signalling proteins, or MAVS proteins for short, that are found on the surface of mitochondria within the cell. This causes the MAVS proteins to form filaments, which have an important role in activating the transcription factors that ultimately lead to the production of the interferons (Figure 1; Hou et al., 2011; Belgnaoui et al., 2011). However, the structure of the filaments formed by the MAVS proteins has long been a mystery. Now, Qiu-Xing Jiang, Zhijian Chen, Xuewu Zhang and co-workers at the UT Southwestern (UTSW) Medical Center and the Janelia Farm Research Campus—with Hui Xu as the first author—have used cryo-electron microscopy to show that these filaments have a novel three-stranded structure (Xu et al., 2014).

**Figure 1.**
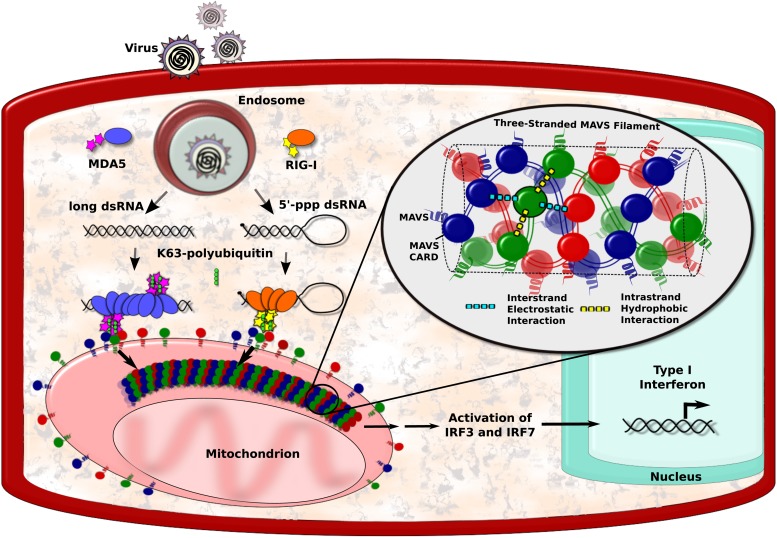
MAVS filaments and activation of the IFN-I pathway. Viral infection introduces foreign RNA molecules into cells (top left), where they are detected by various sensors. A sensor called RIG-I binds to RNA molecules that contain an exposed 5′-triphosphate and terminal dsRNA structure, while MDA5 binds to long dsRNA molecules (Goubau and Deddouche, 2013). The complexes formed by the RNA molecules and the sensors then activate MAVS (mitochondrial antiviral signalling) proteins that are found on the surface of mitochondria. This occurs in the presence of polyubiquitin, which serves to bridge and stabilise the complexes (Jiang et al., 2012). Binding between the complexes and the MAVS proteins occurs at regions on each named CARDs (caspase activation and recruitment domains). In the figure, the RIG-I and MDA5 CARDs are shown as stars, and the MAVS CARDs by the red, blue and green circles. This binding organises the MAVS proteins into filaments to form a signalling platform that activates transcription factors (IRF3 and IRF7), which go on to stimulate the transcription of antiviral type I interferon (IFN-I) genes. Xu et al. report that each MAVS filament contains three intertwined helical strands (shown in red, blue and green). The structure is maintained by interactions between the MAVS CARDs: each CARD in a filament interacts with two other CARDs in the same strand and two CARDs in neighbouring strands. Interactions between strands are hydrophobic (cyan dashed lines), and interactions within a strand are electrostatic (yellow dashed lines).

The caspase activation and recruitment domain (CARD) on a MAVS protein is a bundle of six helix-shaped structures (Potter et al., 2008). The different amino acid residues that form these proteins provide the different regions of the helices with different properties. For example, some regions have electrostatic charges, whereas others are hydrophobic. Xu et al. found that each CARD interacts with four other CARDs—two from the same strand and two from neighbouring strands. The CARDs belonging to the same strand appear to be held together by hydrophobic interactions, while the CARDs from different strands are held together by electrostatic interactions (Figure 1).

Examining cells using super-resolution microscopy revealed that the MAVS filaments are distributed on mitochondria in rod-shaped clusters and average around 400 nm in length. Interestingly, some of these clusters even appeared to bridge mitochondria, which suggests filaments may form from MAVS from multiple mitochondria.

Xu and co-workers then mutated the hydrophobic and charged residues on the interface of CARDs to see how this affected the structure and function of the filaments. They found that this disrupted both filament formation and the activation of interferon, suggesting that these residues play important roles in the interactions within and between strands that keep the filament structure intact and functional.

As Xu et al. note, it is not clear how a RIG-I-RNA or an MDA5-RNA complex triggers the formation of a MAVS filament. Further work should focus on determining the minimum size or number of these complexes that are required to start a filament. In the case of RIG-I, it is known that the length of the double-stranded region of RNA determines the number of RIG-I molecules that bind to the RNA, which consequently dictates the degree of interferon activation. So, it will also be interesting to discover whether the size or the number of activated RIG-I/MDA5 complexes dictates the length or the number of MAVS filaments formed in the cell. Subsequently, does this determine the magnitude of interferon activation?

Finally, what stops the signalling and what happens to the filaments when they are no longer needed? It would seem inefficient in terms of both energy and materials to break down the filaments into their original constituents. Thus, it is possible that other mechanisms such as autophagy, which involves recycling of unnecessary or damaged components of the cell, plays a role in discarding mitochondria containing MAVS filaments. Again, detailed mechanistic studies will be needed to answer these questions.

Despite these outstanding questions, the work of Xu et al. clearly demonstrates that the well-organised structure of MAVS filaments forms the basis of a localised signalling platform that can efficiently recruit and activate molecules in a way that maximises the action of interferon during viral infection.
